# Psychiatric Comorbidities in Hyperacusis and Misophonia: A Systematic Review

**DOI:** 10.3390/audiolres15040101

**Published:** 2025-08-07

**Authors:** Ana Luísa Moura Rodrigues, Hashir Aazh

**Affiliations:** 1 Hospital das Forças Armadas, 1649-020 Lisboa, Portugal; 2 Hashir International Specialist Clinics & Research Institute for Misophonia, Tinnitus and Hyperacusis, London W1W 5PF, UK; info@hashirtinnitusclinic.com

**Keywords:** hyperacusis, misophonia, sound sensitivity, decreased sound tolerance, psychopathology, psychiatric comorbidity

## Abstract

**Background**: The aim of this study was to conduct a systematic review of the research literature on the prevalence of psychiatric comorbidities in patients with hyperacusis and misophonia. **Method**: Four databases were searched: PubMed, PsycINFO, Scopus, and Web of Science (Wis)—last search conducted on the 16th of April 2024 to identify relevant studies. The methodological quality of each study was independently assessed using the JBI Critical Appraisal Checklist. **Results**: Five studies were included for the prevalence of psychiatric comorbidities in hyperacusis, and seventeen studies for misophonia. Among patients with hyperacusis, between 8% and 80% had depression, and between 39% and 61% had any anxiety disorder as measured via a diagnostic interview and/or self-report questionnaires. For misophonia, nine studies provided data on various forms of mood and anxiety disorders, with prevalences ranging from 1.1% to 37.3% and 0.2% to 69%, respectively. **Conclusions**: Although the 22 included studies varied considerably in design and scope, some recurring patterns of comorbidity were noted. However, apparent trends—such as the higher prevalence of mood and anxiety disorders compared to other psychiatric conditions—should be interpreted with caution, as most studies did not comprehensively assess a full range of psychiatric disorders. This likely skews prevalence estimates toward the conditions that were specifically investigated.

## 1. Introduction

Hyperacusis is intolerance to certain everyday sounds, such as domestic noise or noise in public places, which are perceived as too loud or uncomfortable and cause significant distress and impairment in the individual’s life [1]. Tyler et al. (2014) [2] described four categories of hyperacusis comprising (1) loudness hyperacusis, (2) fear hyperacusis, (3) pain hyperacusis, and (4) annoyance hyperacusis. There is some overlap between annoyance hyperacusis and misophonia. Misophonia is characterized by heightened emotional and behavioral reactions (e.g., extreme annoyance, disgust, anger, and avoidance behaviors) to certain sounds with a specific pattern and/or meaning to the individual, such as noises related to eating, lip smacking, sniffling, breathing, clicking sounds, or tapping [3]. Hyperacusis often coexists with tinnitus [4], and it has been associated with a wide range of psychiatric disorders comprising anxiety disorders, post-traumatic stress disorder (PTSD), neuroticism personality traits [5], depression [6,7], and autism spectrum disorders (ASDs) [8]. There are also several studies that suggest that misophonia co-occurs with certain psychiatric disorders, including mood disorders, social phobia, personality disorders with impulsive aggression, intermittent explosive disorder, antisocial personality disorder, obsessive-compulsive disorder (OCD), PTSD, attention deficit hyperactivity disorder (ADHD), ASD, and eating disorders [9,10].

The coexistence of a psychiatric comorbidity can affect the severity of the symptoms and, subsequently, the clinical management of these patients. Despite the psychiatric ill-health of patients with hyperacusis and misophonia being well-known, the occurrence of existing psychiatric comorbidities is not clear and has only been sparsely described [3].

Given this gap, accurate estimates of psychiatric comorbid disorders in both conditions are essential for a better understanding of both disorders and for effective planning, implementation, and evaluation of targeted care interventions. To provide a more comprehensive understanding of such a complex clinical picture, the main aim of this study was to systematically review, identify, collate, analyze, and synthesize the available evidence on the prevalence of psychiatric comorbidities and related factors in patients with hyperacusis and misophonia.

## 2. Methods

This systematic review was conducted and reported according to the Preferred Reporting Items for Systematic Reviews and Meta-analyses (PRISMA) statement [11]. The protocol for this review was registered in the PROSPERO database under record CRD42022347013.

### 2.1. Search Strategy

Systematic searches were conducted by first author (ALR) in cooperation with an academic librarian on the following four bibliographic databases—PubMed, PsycINFO, Scopus, and Web of Science (WoS). Searches were limited to human studies, and no date, language, or country origin restrictions were applied. The search strategy used the terms (Hyperacusis OR Misophonia OR Auditory hypersensitivity OR Auditory Sensitivity OR Noise Sensitivity OR sound sensitivity OR Sound Intolerance OR decreased sound tolerance OR aversive sound OR trigger sound OR Selective Sound Sensitivity Syndrome) AND (Psychiatric symptom OR Psychiatric comorbidity OR Psychiatric disorder OR Mental disorder OR Psychopathology OR Psychiatric Illness OR Mental Illness). A detailed search strategy is provided in Appendix A. To identify further relevant records, the gray literature was explored, and all studies included were subjected to backward and forward citation tracking by hand-searching reference lists and Google Scholar, respectively (initial search conducted on 7th January 2023 and updated search conducted on the 16th of April 2024).

### 2.2. Eligibility Criteria

Studies that report psychiatric comorbidities for at least one psychiatric disorder or if they provided sufficient epidemiologic information (e.g., prevalence data obtained by screening tools) on patients with hyperacusis and patients with misophonia were considered eligible for this review. Studies with mixed samples were considered only if original quantitative data were provided separately for the populations of interest. Inclusion criteria comprised human studies (no age restrictions), particularly observational population-based cross-sectional studies. Other designs that provided data on prevalence for the occurrence of common psychiatric comorbidities were also considered for inclusion, such as longitudinal/cohort studies (prospective and retrospective), case-control studies, case series studies, and randomized clinical trials (RCTs). Qualitative studies, review articles, case reports, case series, letters to the editor, editorials, and opinion pieces were excluded. The primary outcome was the prevalence of any psychiatric comorbidity, ascertained through clinical assessment, including validated assessment methods as standard diagnostic criteria and classification systems (i.e., any version of the DSM or ICD), validated tools, such as self-report scales, clinically structured diagnostic and clinical interviews, such as Mini-international Neuropsychiatric Interview (MINI) and Structured Clinical Interview for DSM (SCID), or proxy-reported disorder-specific questionnaires, and medical records review. Studies were excluded if psychiatric diagnoses were merely self-declared by the participants or in cases in which surveys used non-validated single questions or instruments.

### 2.3. Study Selection and Data Extraction

Identified records were exported to EndNote^TM^ 20.4.1 software, and duplicates were removed. The first author screened titles and abstracts, and an independent reviewer screened a random sample of 25% of all records. Full-text articles were then retrieved and assessed for inclusion by the first author, with 25% of the studies independently analyzed by a second reviewer. All disagreements regarding inclusion were discussed by the first author and the independent reviewer until a consensus was reached or settled by referral to the wider team. The first author extracted data as shown in Table 1 and Table 2. The second reviewer independently cross-checked and validated the extracted data. An updated systematic search was conducted for new studies in April 2024. The first author screened titles and abstracts, and the second author independently screened a random sample of 25% of all the new records. Full-text articles were retrieved and assessed for inclusion by the first author, with all the studies independently analyzed by the second author. The second author independently cross-checked and validated the extracted data.

### 2.4. Quality and Bias Assessment

Methodological quality of each included study was independently assessed by the first author using the Joanna Briggs Institute (JBI) Critical Appraisal Checklist for Prevalence studies, Case-Control studies, and RCTs [12]. The JBI tool for prevalence studies uses nine criteria, the JBI tool for Case-Control Studies uses ten criteria, and the JBI tool for RCTs studies uses thirteen criteria. For each of the 9,10, and 13 checklist items, studies were rated as fully meeting the criterion (‘Y’), being unclear as to whether the criteria were met (‘U’), or not fully meeting the criteria (‘N’). An overall quality rating was assigned to each study (Appendix B). For consistency purposes, the assessment and appraisals of each study were independently checked by an independent reviewer.

### 2.5. Data Synthesis and Analysis

Due to the diversity of methods used to assess hyperacusis, misophonia, and psychiatric comorbidities, and the small number of studies assessing certain specific psychiatric conditions, we did not perform a meta-analysis. Instead, quantitative data were tabulated, and a narrative analysis was conducted.

## 3. Results

**Figure 1:**
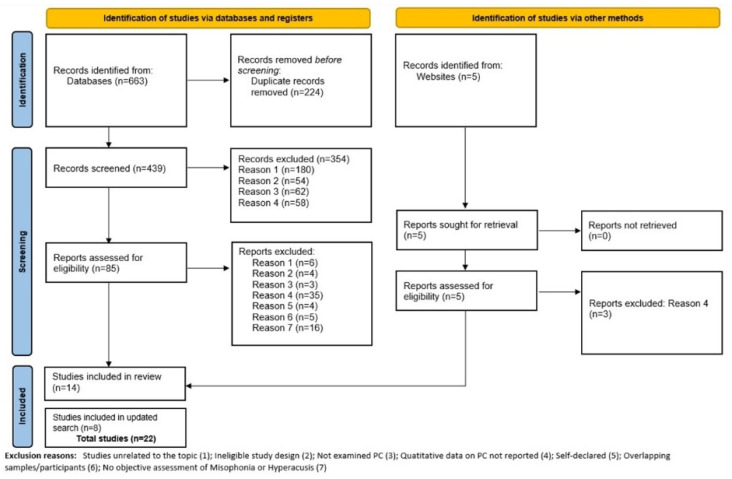
PRISMA flow chart.

The study selection process is shown in the PRISMA flow chart (Figure 1). The initial database search identified 663 records, with an additional 5 studies being identified via the gray literature search, yielding a total of 668 results. After removing duplicates, 444 records were identified. After title and abstract screenings, 90 studies were included for full-text review. From these, 76 articles were excluded for various reasons shown in Figure 1. As a result, 14 studies met the criteria for inclusion, representing 15 data sets (1 study included 2 data sets). We conducted an updated search in April 2024 to include any new studies that emerged since the initial search. Eight new studies met criteria for inclusion, representing in total of twenty-three data sets. Seventeen studies describe the prevalence of psychiatric comorbidities in individuals with misophonia and five in individuals with hyperacusis. Most studies were cross-sectional observational studies, except for three interventional studies [13,14,15]. The studies were conducted in nine countries and represent a total of 6473 participants (4699 with hyperacusis and 1774 with misophonia). The main characteristics of these studies are presented in Table 1 and Table 2. Most of the studies (17) were published within the last 5 years. Psychiatric comorbidities were assessed using clinical diagnostic interviews in eighteen studies [5,9,13,14,15,16,17,18,19,20,21,22,23,24,25,26,27,28] and/or self-report questionnaires in four studies [29,30,31,32]. Hyperacusis was assessed by self-reported measures in two studies [19,29], by single question in one study [31], by psychiatric screening in one study [28] and using a ULL based criteria in one study [5]. Misophonia was assessed by self-report questionnaires in fifteen studies [9,13,14,16,17,18,21,22,23,25,26,27,30,32], by psychiatrist screening in one study [15] and by patient self-report in one study [20].

The quality of 8 studies was rated as high [19,20,21,26,27,28,30,31], while 14 were rated as having moderate quality [5,9,13,14,15,16,17,18,29,32]. Results for study quality are presented in Appendix B. Considering the broad criteria against which risk of bias can be assessed in prevalence studies, we deemed the risk of bias across these studies to be moderate to high, as samples were not likely to be representative of the general population and some studies used screening questionnaires rather than diagnostic interviews to assess for mental health conditions, potentially introducing issues of reliability and validity.

### 3.1. Psychiatric Comorbidities in Hyperacusis

#### 3.1.1. Mood Disorders

Four studies included in the review provided prevalence data for depression and/or depressive symptomology ranging from 8% to 80% (Table 3). The prevalence of depression was measured via a diagnostic interview in one study [19] and via self-report questionnaires in three studies [5,29,31]. Jüris et al. [5] used ULL-based criteria to diagnose hyperacusis, but the other three studies used self-report questionnaires.

#### 3.1.2. Anxiety Disorders

All five studies reported prevalence data for co-occurring anxiety and obsessive-compulsive and related disorders among patients with hyperacusis, ranging from 3% to 61% (Table 4). Anxiety disorders as a broad category were the most frequently assessed disorder among the studies, and they had the highest prevalence rate (39% to 61%) as measured via diagnostic interview [5,19,28] or using self-report questionnaires [29,31].

#### 3.1.3. Other Psychiatric Disorders

Substance abuse disorders and eating disorders were evaluated in a single study [5]. Alcohol disorder was present in 5% of participants with hyperacusis and eating disorders in 2%. Somatoform disorder was present in 22% of patients with hyperacusis [19] (Table 1).

### 3.2. Psychiatric Comorbidities in Misophonia

#### 3.2.1. Mood Disorders

Fourteen studies provided data on the prevalence of various forms of mood disorders among patients with misophonia, ranging from 1.1% to 37.3% (Table 5). The most frequently assessed type of mood disorder was major depressive disorder (MDD), which was reported in ten studies with prevalences between 1.1% and 18.4%. There was a large gap between the two studies that reported suicidal ideations: 20% [26] versus 1.92% [18]. The discrepancy is likely to be related to their sample characteristics, as the prevalence of MDD was smaller in the study by Erfanian, Kartsonaki, and Keshavarz [18] compared to the study by Siepsiak, Rosenthal, Raj-Koziak, and Dragan [26], 9.61% vs. 11.9%, respectively. All the studies used an interview method for psychiatric diagnosis, except [32], which used a self-report screening scale.

#### 3.2.2. Anxiety and Trauma Related Disorders

Fifteen studies provided data on the prevalence of co-occurring anxiety and trauma-related disorders in patients with misophonia, with prevalence rates ranging between 0.2% and 69% (Table 6). Generalized anxiety disorder (GAD) (prevalence between 1% and 36.2%) and social anxiety (prevalence between 1.2% and 30.9%) were the most frequently assessed disorders across the studies. All of the studies used an interview method for psychiatric diagnosis, except [32], which used a self-report screening scale.

#### 3.2.3. Obsessive-Compulsive Related Disorders

Thirteen studies provided data on the prevalence of co-occurring obsessive-compulsive related disorders in misophonia (Table 7). OCD was the most frequently assessed disorder, with prevalence rates ranging from 2.1% to 39.8% [9,15,16,18,21,22,23,24,25,26,32].

#### 3.2.4. Eating Disorders

Seven studies included the diagnosis of co-occurring eating disorders. Bulimia was the most frequently assessed eating disorder, with prevalence rates from 1.4% to 11.9% [16,18,24,26] (Table 8). Anorexia was the eating disorder with the highest prevalence rate (between 3.1% and 14.9%) assessed in three studies [16,18,26]. All the studies used a diagnostic interview method.

#### 3.2.5. Substance Use Disorders

Five studies included the diagnosis of substance use disorders (Table 9). This included the diagnosis for alcohol, cannabis, cocaine, and other (not specified). The most frequently reported disorder was alcohol dependency, assessed in four studies (prevalence rates between 1.0% and 11%) [13,18,22,24]. All the studies used diagnostic interview methods.

#### 3.2.6. Neurodevelopmental Disorders

Twelve studies provided data on the prevalence of neurodevelopment disorders (Table 10). ADHD was the disorder most assessed across studies [13,15,16,17,21,22,23,24,25,27], with prevalence rates varying from 1.7% to 21%. Autistic disorder had the highest prevalence rate of 21.4% assessed in a single study using a self-report screening scale [30]; therefore, results should be interpreted with caution. All the studies used an interview method for psychiatric diagnosis, except for [30], which used a self-report screening scale.

#### 3.2.7. Personality Disorders

Four studies assessed the presence of personality disorders among patients with misophonia (Table 11). All four studies [9,22,24,25] provided prevalence data for obsessive-compulsive personality disorder (OCPD), with prevalence rates ranging from 2.4% to 52.4%. All the studies assessed personality disorders using a diagnostic interview method.

#### 3.2.8. Other Psychiatric Disorders

Four studies provided data for hypochondriasis (prevalence between 0.9% and 2.6%) [13,20,22,24,25]. Two studies [18,22] provided data on psychotic disorders, with one of them [18] assessing the presence of psychotic disorders as a broad diagnostic category (1.92%) and one study [22] assessing schizophrenia (0.2%). All studies used a diagnostic interview method.

## 4. Discussion

This systematic review aimed to examine the occurrence of the full range of psychopathology in individuals suffering from hyperacusis and misophonia. As far as the authors are aware, this is the first review to systematically summarize the occurrence of psychiatric comorbidities in both conditions. Collectively, the evidence base suggests that both conditions are associated with greater psychopathology and with a wide range of psychiatric disorders, not with one specific disorder. Most of the studies were published within the last 5 years, demonstrating an increasing interest in recent times. Most of the studies included in this review were conducted predominantly in Europe and the USA. All the studies included clinical and community-sourced samples, and a range of methods were used to assess psychiatric comorbidities, such as standardized structured interviews and self-report screening instruments. The quality of the studies was variable and rated using a standardized assessment tool. The quality of eight studies was rated as high, while fourteen were rated as having moderate quality. However, even among the high-quality studies, there were important limitations in case identification. For example, Smit et al. (2021) [31] assessed hyperacusis using a single self-report question rather than a validated questionnaire, limiting confidence in the accuracy of classification. Likewise, Guetta et al. (2024) [20] relied on patient self-report for a misophonia diagnosis without applying standardized diagnostic criteria or structured assessment tools. These methodological issues constrain the interpretability and comparability of prevalence estimates across studies, and caution is warranted when drawing conclusions from such data. The most frequently assessed disorder among patients with hyperacusis and misophonia was depression. This is consistent with the known fact that depression is a leading burden of psychiatric comorbidity worldwide [33].

Seventy-five percent of the studies (three out of four studies) that assessed depression among patients with hyperacusis reported prevalence rates of above 40% in this population. Eighty percent of studies (eight out of ten) that assessed major depressive disorders (MDDs) among patients with misophonia reported prevalence rates between 6% and 19%, and the remaining 20% of studies reported prevalence rates of below 5% for MDD. Unlike the hyperacusis studies, which about 50% used self-report questionnaires to diagnose depression, all the misophonia studies (10 out 10) that reported MDD used a clinical interview with a mental health professional as the basis for diagnosing depression. This can partly explain the difference between prevalence rates reported for depression between patients with hyperacusis and misophonia. However, given the large gap between the reported prevalence rates, it is likely that patients with hyperacusis exhibit more depressive symptoms compared to patients with misophonia. This is consistent with a recent study on patients who sought help from a specialist center in the UK for distressing misophonia, hyperacusis, and tinnitus. They reported that 50% of patients with misophonia, compared to 100% of patients with hyperacusis, had abnormal scores on PHQ-9, indicating the presence of depression [34]. Future studies should further compare the mechanism that produces depression between patients with hyperacusis and misophonia and their mediating and moderating factors [35].

All the studies (five out of five) on hyperacusis reported prevalence rates above 40% for anxiety disorders. However, 21% of the studies (3 out of 14 studies) that assessed anxiety among patients with misophonia reported prevalence rates of above 40% for any type of anxiety disorders. Therefore, it seems that anxiety is also more prevalent among patients with hyperacusis compared to misophonia. This conclusion needs to be interpreted with caution, given the differences in methodologies used and study populations between studies on hyperacusis and misophonia. It is noteworthy that OCD was commonly reported across misophonia studies (n = 11), with prevalence rates varying from 2.1% to 39.8%. In fact, some authors suggested that misophonia could be categorized within the obsessive-compulsive spectrum disorders [25].

Although there is a significant variation in prevalence rates between both conditions, the patterns of comorbidity observed are consistent, with mood and anxiety disorders appearing to be more prevalent than other types of comorbidities. Overall, the result of this systematic review suggests that prevalence rates for depression and anxiety among patients with hyperacusis and misophonia are much higher than the current rates of depression and anxiety in the general population, which are estimated to range from 3.4% to 7.5 [35]. These rates increased significantly during the COVID-19 pandemic, with data showing an approximately 30% rise in depression and anxiety disorders, reflecting the growing burden of psychiatric comorbidities globally (Santomauro et al., 2021) [34]. 

### 4.1. Future Directions

The current research evidence relies on cross-sectional data and does not allow the examination of how hyperacusis and misophonia and the range of psychopathology interact and evolve over time. Future longitudinal studies with large and more diverse participant groups will be crucial to unravel causal relationships and better understand the evolution of these disorders. Additionally, incorporating standardized assessment tools and diagnostic criteria not only to assess psychiatric disorders but also for hyperacusis and misophonia will enhance the comparability and reliability of findings across different studies. Finally, this review provides the best current estimate of psychiatric comorbidities, but it is possible that the prevalence is under- or overestimated in patients with hyperacusis and misophonia. This lack of clarity regarding the prevalence of psychiatric comorbidities in these two distinct populations does not diminish the importance of diagnosing and treating psychiatric comorbidities in these patients, who need mental health care incorporated in their management plans.

### 4.2. Limitations

Although this study provides an updated and comprehensive overview of the current body of publications—allowing for the identification of future research avenues—the primary studies included in our systematic review have significant limitations that may affect our findings. First, the variability of the assessment methods. Secondly, and contrarily to misophonia studies, several psychiatric comorbidities were not assessed in hyperacusis studies (e.g., ADHD, autism spectrum disorders, personality disorders), and therefore, it is challenging to make comparisons between both conditions. The assessment tools used could explain why these comorbidities are absent from the studies. For example, the PHQ-9 and DASS-21 are not diagnostic tools (i.e., they cannot be used to diagnose depression); rather, they are both screening tools that may identify some symptoms of depression that can assist professionals in making onward referrals to mental health services. As previously mentioned, these various self-report questionnaires are likely to contribute to the heterogeneity in findings and may influence prevalence rates. For depression and anxiety, while studies using interview methods yielded a reasonably broad range of prevalence rates, studies using self-report screening measures (i.e., PHQ-9, DASS-21, GAD-7) generally yielded higher prevalence rates for both disorders [29,31]. Therefore, prevalence estimates generated by studies that only use self-report screening tools should be interpreted with caution.

Similarly, the reliability of the prevalence rates may also be related to the variability of the sample sizes, convenience sampling associated with clinic-based studies, and demographic characteristics. For example, one study included 15 participants [30] and another included 775 participants [31]. Most of the studies were also primarily comprised of female participants, and in four studies, there was no data on the gender ratio (related to the final sample). Although every effort was put into obtaining further information from corresponding authors, in cases where there was no response, we were also limited by the amount of demographic data available.

## Figures and Tables

**Table 1:** Main characteristics of the 5 hyperacusis-related primary studies included in the review.

Study	Country	Study Design	Study Setting	Aim(s)	Initial Sample Size ^a)^	Hyperacusis Sample Size	Comorbid Tinnitus	Hyperacusis Diagnosis	Hyperacusis Sample Age (Mean, SD)	Gender: Female (n, %)	Exclusion Criteria	Psychiatric Diagnostic Method(s) ^b)^	Psychopathology Quantitative Data
Aazh and Moore (2017)	United Kingdom	Observational cross-sectional questionnaire-based study	clinical-referral sample	To determine the relevance and applicability of psychological questionnaires to patients seeking help for tinnitus and/or hyperacusis	145	37	Not reported	Hyperacusis questionnaire (HQ) score 26	Not specified for the hyperacusis sample	Not specified for the hyperacusis sample	Not specified	HADS (depression and anxiety); GAD-7 (anxiety); SHAI (Health anxiety); Mini-SPIN (social anxiety); OCI-R (OCD); PDSS-SR (panic disorder); PHQ-9 (depression); PSWQ-A (GAD)	Mood disorders—depression: 47% Anxiety disorders—anxiety: 61%; health anxiety: 56%; GAD: 53%; social anxiety: 51%; OCD: 37%; panic disorder: 37%
Goebel and Floetzinger (2008)	Germany	Cross-sectional observational pilot study	Clinical inpatient sample	To investigate the type and degree of psychiatric comorbidity in patients with chronic tinnitus with and without hyperacusis	163	95	100%	Structured Tinnitus Interview (STI) items; STI Hyperacusis scale	Not specified for the hyperacusis sample	Not specified for the hyperacusis sample	More than moderate hearing loss (better ear), severe recruitment, conflicts with insurances or desire to gain retired status; substance abuse, Meniere’s disease, middle ear muscle dysfunction, Williams syndrome, Ramsay-Hunt syndrome, facial paralysis, episodes of hypoglycaemia, hyperthyreosis, phaeo-chromocytom, carcinoids, epilepsy; psychotic or schizoaffective disorders and certain mental disorders	Checklists for ICD-10; BDI (depression); BAI (anxiety); WI (health anxiety)	Depression: 58% anxiety disorder: 39%; somatoform disorder: 22%
Jüris et al. (2012)	Sweden	Not specified	Clinical outpatient sample	To investigate psychiatric comorbidity based on DSM-IV and personality traits in patients with hyperacusis	81	62	79%	Uncomfortable loudness levels (ULLs); hearing levels	40.2 (12.2)	47 (76%)	Tinnitus as the primary audiological problem; Hearing levels < 40 dB	MINI for DSM-IV and ICD-10	Mood Disorders— any affective disorder: 15%; MDD: 8% dysthymic disorder: 8% Anxiety Disorders—any anxiety disorder: 47%; social phobia: 23%; GAD: 16%; agoraphobia: 15%; OCD: 10%; panic disorder: 6%; PTSD: 3% Other disorders— any alcohol abuse: 5%; eating disorders: 2%
Smit et al. (2021)	Australia	Prospective population-based study	Non-clinical community sample	To determine the prevalence of hyperacusis and its relation with hearing, general and mental health in a population-based study	5107	775	40.5%	Single screening question: “Do you consider yourself sensitive or intolerant to everyday sounds”	58.83 (5.82)	452 (58.3%)	Institutionalized adults	PHQ-9 (used against DSM-IV criteria and following severity cut-off scores) DASS-21	DASS-21—Depression (overall severity): 41%; anxiety (overall severity): 43% PHQ-9—Depression (overall severity): 79.9%; MMD (DSM-IV): 5.7%; other depressive disorder (DSM-IV): 4.5%
Theodoroff et al. (2024)	USA	Retrospective observational study	Clinical inpatient and outpatient samples	To estimate the prevalence of hyperacusis diagnosis in treatment-seeking veterans	5,744,670	3724	73%	ICD billing codes (ICD-9 and ICD-10)	Not specified	839 (22.5%)	Not specified	Medical records review	PTSD: 49%

**^a)^** Refers to sample size before extracting the cases of hyperacusis **^b)^** Also includes the diagnostic interviews and screening tools used BAI: Beck Anxiety Inventory; BDI: Beck Depression Inventory; DASS: Depression Anxiety Stress Scale; dB: decibels: DSM: Diagnostic and Statistical Manual; GAD: generalized anxiety disorder; HADS: Hospital Anxiety and Depression Scale; ICD: International Classification of Diseases; MDD: major depressive disorder; MINI: Mini International Neuropsychiatric Interview; **n**: number of female participants; OCD: obsessive-compulsive disorder; OCI: Obsessive Compulsive Inventory; PDSS: Panic Disorder Severity Scale; PHQ: Patient Health Questionnaire; PSWQ: Penn State Worry Questionnaire; PTSD: post-traumatic stress-disorder; SD: standard deviation; SHAI: Short Health Anxiety Inventory; SPIN: Social Phobia Inventory WI: Whiteley Index.

**Table 2:** Main characteristics of the 17 misophonia-related primary studies included in the review.

Study	Country	Study Design	Study Setting	Aim(s)	Initial Sample Size ^a)^	Misophonia Sample Size	Misophonia Diagnosis	Misophonia Sample Age (Mean, SD)	Gender: Female (n, %)	Exclusion Criteria	Psychiatric Diagnostic Method(s) ^b)^	Psychopathology Quantitative Data
Abramovitch et al. (2024)	USA	Case-control observational study	Community sample (students)	To conduct the first neuropsychological study of misophonia in the context of cognitive function.	275	32	Misophonia Questionnaire (MQ)	21.06 (2.45)	19 (59.40%)	Age< 18 or > 65; any history of major neurological conditions; uncorrected vision problems; non-English speakers	MINI 7.0 for DSM-5	Mood disorders—MDD: 3.10% Anxiety and Obsessive-Compulsive and Related Disorders—GAD: 3.1%; social anxiety disorder: 3.1%; panic:6.3%; agoraphobia: 3.2%; OCD: 9.4%; PTSD: 3.1%; any anxiety disorder: 18.8% Eating disorders—anorexia nervosa:3.1%; Bulimia nervosa: 3.1%; binge eating: 3.1%; any eating disorder: 6.3% Other disorders—ADHD: 15.6%; substance abuse 9.4%; any current DSM disorder: 31.30%
Begenen et al. (2023)	Turkey	Cross-sectional observational study	Clinical-referral sample (psychiatric clinic)	To compare patients with OCD with and without misophonia in terms of sociodemographic data, clinical features, and executive functions.	77	39	AMC 2013 criteria (Schröder et al.);	23.9 (6)	23 (56%)	Presence of dementia, psychotic disorder, BD, SUD, suicide ideations, intellectual disability; chronic internal/neurological conditions; moderate to- severe depressive disorder and patients who could not complete neuropsychiatric tests	SCID-5 for DSM-5	Mood disorders—depressive disorder: 17.9%; dysthymic disorder:5.1%; premenstrual dysphoric disorder:15.4% Anxiety and Obsessive-Compulsive and Related Disorders—GAD: 12.8%; social anxiety disorder: 17.9%; panic:10.3%; agoraphobia: 25.6%; specific phobia:12.8%; any obsessive-compulsive: 35.9%; any anxiety disorder: 59%; body dysmorphic disorder: 2.6%; excoriation: 5.1%; hoarding: 12.8%; onychophagia:17.9% Neurodevelopmental disorders ADHD: 20.5%; tic disorder: 5.1%; Other disorders—hypochondria: 2.6%; binge eating: 15.4%; gambling addiction: 5.1%; any psychiatric disorder: 82.1%
Cassiello-Robbins et al. (2021)	USA	Cross-sectional correlational study	Community sample	To examine the relationship between symptoms of misophonia and psychiatric diagnoses in a sample of community adults, using semi-structured diagnostic interviews	NA	49	Misophonia Questionnaire (MQ)	27.02 (8.75)	42 (85.7%)	Age ≤ 18; current mania or psychotic disorder	SCID-I for DSM-IV (Axis I disorders); SCID-II for DSM-IV (Axis II—personality disorders); BDI-II (depressive symptoms); BAI (anxiety symptoms)	Mood disorders—MDD: 18.4% Anxiety and Obsessive-Compulsive and Related Disorders—GAD: 32.7%; social anxiety disorder: 10.2%; OCD: 6.1%; PTSD: 18.4% Personality Disorders—APD: 8.2%; BPD: 12.2%; OCPD: 10.2%
Erfanian et al. (2019)	Iran	Cross-sectional observational study	Clinical-referral sample (psychiatric clinic)	To examine the prevalence of psychiatric symptoms in clinically diagnosed misophonia sufferers, the association between misophonia severity and psychiatric symptoms, and gender	NA	52	AMC 2013 criteria (Schröder et al.); Amsterdam Misophonia Scale (A-MISO-S)	45.27 (16.23)	30 (58%)	A-MISO-S score ≤ 4; co-morbid tinnitus, hyperacusis, diplacusis, hearing impairment; patients taking antidepressants or anxiolytics in the last 2 week	MINI 5.0.0	Mood Disorders—MDD: 9.61%; dysthymia: 5.76%; suicidality: 1.92% Anxiety and Obsessive-Compulsive and Related Disorders—PTSD: 15.38%; OCD: 11.53%; social phobia: 5.76%; panic: 1.92%; agoraphobia; 1.92%; GAD: 1.92% Eating Disorders—anorexia: 9.61%; bulimia: 7.69% Substance use disorders—alcohol dependence: 3.84%; alcohol abuse: 3.84%; substance dependence: 1.92%; substance abuse 1.92% Other disorders—psychotic disorders: 1.92%
Frank and McKay (2019)	USA	RCT (in-progress)	Community sample	To assess effect of inhibitory learning as a treatment paradigm for misophonia.	58	18	Misophonia Questionnaire (MQ)	34.94 (10.95)	Not reported	Not Specified	SCID-5 for DSM-5; DASS-21	Mood disorders—MDD: 22%; PDD: 11%; premenstrual dysphoric disorder: 6% Anxiety Disorders and Obsessive-Compulsive and Related Disorders panic disorder: 6%; GAD: 17%; specific phobia: 11%; social anxiety disorder: 11%; agoraphobia: 11%; OCD: 11%; excoriation: 6% Other disorders—adult ADHD: 6%; alcohol use disorder: 11%; hypersomnolence disorder: 11%
Guetta et al. (2024)	USA	Cross-sectional correlational study	Community sample	To understand the relationships among misophonia, stress, and trauma in a community sample and to examine mechanisms of trauma and stress-related sequelae that contribute to misophonia severity	N/A	143	Self-reported.	36.88 (18.84)	100 (69.9%)	Current psychotic disorder, mania, and anorexia nervosa	SCID-5 for DSM-5, research version; PCL-5 for DSM-5	Trauma and stressor-related disorder—PTSD: 3.5%; adjustment disorder 2.8%; trauma disorder OS: 5.6%;
Guzick et al. (2023)	USA	Cross-sectional observational study	Community sample	To describe the clinical phenomenology of youth with misophonia and to evaluate associations between misophonia severity and psychiatric symptoms, as well as quality of life	152	102	Amsterdam Misophonia Scale (A-MISO-S)	13.7 (2.5)	69 (68%)	A-MISO-S score ≤ 10; unable to schedule appointments	MINI-KID	Mood disorders—MDE: 15% Anxiety and Obsessive-Compulsive and Related Disorders—GAD: 27% social anxiety disorder: 30% OCD: 8% Neurodevelopmental disorders ADHD: 21%; tic disorder 13% At least one anxiety disorder: 56%
Jager et al. (2020)	Netherlands	Cross-sectional observational study	Clinical-referral sample (outpatient psychiatric university clinic)	To determine whether GP referred subjects with misophonia-like symptoms actually suffered from misophonia using a psychiatric interview, and to determine phenomenology, comorbidity, and demographics of a large misophonia sample	779	575	AMC 2013 criteria (Schröder et al.); Amsterdam Misophonia Scale (A-MISO-S); Amsterdam Misophonia Scale—Revised (A-MISO-R)	34.17 (12.22)	399 (69%)	Primary autism spectrum conditions; primary AD(H)D; a primary diagnosis on Axis II; subjects without a DSM-IV diagnosis	MINI-Plus; SCID-II for DSM-IV (Axis II—personality disorders); HAM-D; HAM-A; Autism Spectrum Quotient (AQ)	Mood disorders—MDD: 6.8%; dysthymic disorder: 1.7%; BD II: 0.7%; BD I: 0.5%; depressive disorder NOS: 0.3% Anxiety and Obsessive-Compulsive and Related Disorders—OCD: 2.8%; PTSD: 1.7%; social phobia: 1.2%; GAD: 1.0%; specific phobia: 1.0%; panic disorder with agoraphobia: 0.9%; separation anxiety disorder: 0.2%; anxiety disorder NOS: 0.2%; trichotillomania or excoriation disorder: 1.9% Neurodevelopmental disorders -autistic disorder: 1.2%; pervasive developmental disorder NOS: 1.2%; attention deficit disorder: 3.3%; ADHD: 1.7%; ADHD combined type: 0.3%; tic disorder NOS: 0.5%; chronic motor or vocal tic disorder: 0.5%; Tourette syndrome: 0.3%; tic disorder: 0.2%; stuttering: 0.2% Substance use disorders—alcohol dependence: 0.7%; cannabis or dependence on sedatives: 0.5%; abuse of alcohol: 0.3% Personality disorders—OCPD: 2.4% BPD: 1.7%; avoidant PD: 0.5%; dependent PD: 0.2%; antisocial PD: 0.2% Other disorders—eating disorder NOS: 0.7%; intermittent explosive disorder: 0.2%; hypochondria: 0.9%; undifferentiated somatoform disorder: 0.5%; schizophrenia: 0.2%
Lewin et.al (2023)	USA	Cross-sectional observational study	Community Sample	To examine the clinical characteristics of youth with misophonia.	47	46	Misophonia assessment interview (MAI; Lewin, 2020;	13.2 (2)	32 (69.6%)	Past treatment for emotional disorders or misophonia; Active psychotic disorder, BD, eating disorder, alcohol/SUD, intellectual disability, or suicidal ideation	ADIS-5	Mood Disorders—MDD: 8.5%; dysthymia:4.3% Anxiety and Obsessive-Compulsive and Related Disorders—OCD: 2.1%; PTSD: 6.4%; GAD: 36.2%; specific phobia: 12.9%; separation anxiety disorder: 2.1%; Social anxiety: 29.8%; any anxiety disorder: 59.6% Neurodevelopmental disorders—autistic disorder: 4.3%; ADHD: 14.9%; tic disorder: 6.4%; Other: oppositional defiant disorder:8.5%
Neacsiu et al. (2024)	USA	Intervention Study	Community Sample	To test differences between adults with misophonia and clinical controls on HF-HRV, SCL, SCR, and self-reported distress during passive listening and regulation of aversive and misophonic sounds. to examine whether misophonia interventions should target reduction of sound reactivity or improvement in emotion regulation.	1341	29	Misophonia Questionnaire (MQ)	29.59 (9.79)	26 (89.66%)	Current or past history of mania or psychosis; IQ < 70; not medically cleared for TMS or fMRI; going to jail in the next 2 months; pregnant; high risk of suicide; moderate/severe current alcohol or substance dependence; unable to attend for 3 visits	SCID-5, SCID-PD	Mood disorders: 13.8% Anxiety disorders: 69% Obsessive compulsive disorders: 6.9% Stress disorders: 10.3% Impulse control disorders: 6.9% Eating disorders: 3.4%
Rinaldi et al. (2022)	United Kingdom	Case-control observational study	Community sample	To investigate what traits might contribute to the profile of people with misophonia, particularly traits typically associated with autism	379	126	Sussex Misophonia Scale (SMS)	30.32 (17.21)	92 (73%)	Self-declared misophonics who did not pass the diagnostic threshold	Autism Spectrum Quotient (AQ)	Autism clinical significant threshold: 21.4%
				To examine whether adolescents with misophonia already show autism-related traits, similar to adults	142	15 (12 with valid AQ)	Sussex Misophonia Scale for Adolescents (SMS-As)	Girls: 11.67 (1.32); Boys: 11 (0.89)	9 (60%)	Not specified	Autism Spectrum Quotient for Adolescents (AQ-Adolescents)	Autism clinical significant threshold: 12.5%
Rosenthal et al. (2022)	USA	Cross-sectional correlational study	Community sample	To improve the phenotypic characterization of misophonia by investigating its psychiatric and medical health correlates in adults	210	207	Misophonia Questionnaire (MQ)	35.72 (12.49)	154 (74.4%)	MINI 7.0.2. criteria for a current psychotic disorder, mania, anorexia; inability to read English	SCID-5 for DSM-5, research version; SCID-5-PD for DSM-5 personality disorders	Mood Disorders—MDD: 6.8%; BD I: 0.5%; BD II: 0.5%; PDD: 7.7%; premenstrual dysphoric disorder: 1.9%; depressive disorder OS: 1.4% Anxiety and Obsessive-Compulsive and Related Disorders—GAD: 24.6%; panic: 2.9%; social anxiety: 30.9%; agoraphobia: 10.1%; specified phobia: 13.5%; OCD: 8.2%; PTSD: 2.9%; body dysmorphic disorder: 0.5%; excoriation: 5.3%; hoarding: 1.9%; trichotillomania: 1.9% Substance use disorders—alcohol: 1.9%; cannabis: 1.4%; stimulants/cocaine: 0.5%; other SUD: 0.5% Eating disorders—bulimia nervosa: 1.4%; binge eating: 0.5%; eating disorder OS: 2.9% Neurodevelopmental disorders ADHD: 17.9%; adult ADHD: 14.5%; ASD: 2.4%; tic disorder: 1.9%; stuttering: 0.5%; specific learning disorder: 0.5%; speech sound disorder: 3.9% Personality disorders—OCPD: 5.8%; avoidant PD: 2.9%; BPD: 2.9%; dependent PD: 0.5%; narcissistic PD: 0.5%; paranoid PD: 0.5%
Schröder et al. (2013)	Netherlands	Cross-sectional observational study	Community sample (recruited via hospital website)	To describe the clinical symptomatology of misophonia, discuss the classification of symptoms, propose diagnostic criteria, and introduce A-MISO-S	NA	42	Amsterdam Misophonia Scale (A-MISO-S)	Not reported (adult sample)	20 (47.6%)	Not specified	SCID-II for DSM-IV (Axis II) —personality disorders); HAM-D (depression level); HAM-A (anxiety symptoms)	Mood Disorder—dysthymic disorder: 7.1% Anxiety and Obsessive-Compulsive and Related Disorders—OCD: 2.4; panic disorder: 2.4%; excoriation: 2.4%; trichotillomania: 4.8% Neurodevelopmental disorders—ADHD: 4.8%; Tourette syndrome: 4.8% Personality disorders—OCPD: 52.4% Other disorders—hypochondria: 2.4%
Schröder et al. (2017)	Netherlands	Intervention study (open-label trial)	Clinical-referral sample	To determine the efficacy of CBT and to investigate if clinical or demographic characteristics predicted treatment response	NA	90	Psychiatrist screening	35.8 (12.2)	65 (72%)	Presence of substance dependence, BD, ASD, or psychotic disorders	Psychiatrist screening	Mood Disorders—MDD: 1.1%; dysthymic disorder: 2.2%; BD II: 1.1% Anxiety and Obsessive-Compulsive and Related Disorders—OCD: 3.3%; excoriation: 5.5%; body dysmorphic disorder: 1.1% Neurodevelopmental disorders— ADHD: 4.4%; Tourette syndrome: 2.2% Other disorders—bulimia/anorexia: 3.3%; hypochondria: 1.1%
Siepsiak et al. (2022)	Poland	Cross-sectional observational study	Community sample (students)	To examine the psychiatric and audiologic features of misophonia, by comparing three groups (a misophonia group, an audiologically healthy control group and a clinical control group)	156	71	AMC 2013 criteria (Schröder et al.); MisoQuest	31.04 (8.79)	64 (90.1%)	Participants with heart diseases, pacemakers, facial hair, or a current substance use disorder	MINI 7.0.2 for DSM-5 and ICD-10	Mood Disorders—MDE: 37.3%; MDD: 11.9%; BD I: 9.0%; BD I with psychotic features: 4.5%; BD II: 1.5%; suicidality: 20.0%; suicidal behavior: 3.0% Anxiety disorders—panic disorder: 19.4%; GAD: 14.9%; social anxiety disorder: 13.4%; PTSD: 11.9%; OCD: 6.0%; agoraphobia: 4.5%; Eating Disorders—anorexia: 1.5%; Bulimia: 1.5%
Siepsiak et al. (2023)	Poland	Case-control observational study	Community sample	To evaluate preliminary findings from previous research on misophonia within a Polish sample of children and adolescents and develop new hypotheses to test in further studies.	N/A	45	Amsterdam Misophonia Scale (A-MISO-S)	13.1 (3)	68.2%	Diagnosis of ASD, intellectual disability, serious somatic illness, hearing loss, and serious sight impairment		Neurodevelopmental disorders—ADHD: 2.4%; ADHD combined type: 7.1%; ADD:16.7%; tic disorder: 18.2%; Other: OCD: 13.6%
Yektatalab et al. (2022)	Iran	Cross-sectional descriptive observational study	Community sample	To determine the prevalence of misophonia and its relationship with obsessive-compulsive disorder, anxiety, and depression in undergraduate students	390	93	Misophonia Questionnaire (MQ)	Not reported (undergraduate sample)	Not specified for the misophonia sample	Self-reported hearing diseases	Maudsley Obsessive-Compulsive Inventory; BAI (2nd Ed); BDI (2nd Ed)	Mood Disorders—depression: 9.7% Anxiety and Obsessive-Compulsive and Related Disorders—OCD: 39.8%; anxiety: 8.6%

**^a)^** Refers to sample size before extracting the cases of misophonia **^b)^** Also includes the diagnostic interviews and screening tools used. ADHD: attention deficit hyperactivity disorder; APD: avoidant personality disorder; ASD: autism spectrum disorder; BAI: Beck Anxiety Inventory; BD: bipolar disorder; BDI: Beck Depression Inventory; BPD: borderline personality disorder; CBT: Cognitive Behavioral Therapy; DASS: Depression Anxiety Stress Scale; DSM: Diagnostic and Statistical Manual; GAD: generalized anxiety disorder; GP: general practitioner; HAM-A: Hamilton Anxiety Rating Scale; HAM-D: Hamilton Depression Rating Scale; MDE: major depressive episode; MDD: major depressive disorder; MINI: Mini International Neuropsychiatric Interview; n: number of female participants; NA: not applicable; NOS: not otherwise specified; OCD: obsessive-compulsive disorder; OCPD: obsessive-compulsive personality disorder; OS: other specified; PD: personality disorder; PDD: persistent depressive disorder; PCL-5: Posttraumatic Stress Disorder Checklist; PTSD: post-traumatic stress-disorder; RCT: randomize controlled trial; SCID: Structured Interview for DSM; SD: standard deviation; SUD: substance use disorder; USA: United States of America.

**Table 3:** Prevalence of comorbid mood disorders in hyperacusis.

	Mood Disorders		
	Depression	Dysthymic Disorder	Any Affective Disorder
**Aazh and Moore (2017) ^b^** **(*N* = 37)**	47% *	-	-
**Goebel and Floetzinger (2008) ^a^** **(*N* = 95)**	64%	-	-
**Jüris et al. (2012) ^a^** **(*N* = 62)**	8%	8%	15%
**Smit et al. (2021) ^b^** **(*N* = 775)**	80% * 41% **	-	-

^a^ Prevalence of mood disorders assessed using a diagnostic interview method. ^b^ Prevalence of mood disorder assessed using a self-report screening scale. * Data obtained using PHQ-9. ** Data obtained using DASS21. (-) Mood disorder not assessed.

**Table 4:** Prevalence of comorbid anxiety disorders in hyperacusis.

	Anxiety Disorders						
	Anxiety Disorder	Generalized Anxiety Disorder	Panic Disorder	Social Anxiety Disorder	Agoraphobia Disorder	OCD	PTSD
**Aazh and Moore (2017) ^b^** **(*N* = 37)**	61%	53%	37%	51%	-	37%	-
**Goebel and Floetzinger (2008) ^a^** **(*N* = 95)**	39%	-	-	-	-	-	-
**Juris et al. (2012) ^a^** **(*N* = 62)**	47%	16%	6%	23%	15%	10%	3%
**Smit et al. (2021) ^b^** **(*N* = 775)**	42.9%	-	-	-	-	-	-
**Theodoroff et al. (2024) ^a^** **(*N* = 3724)**	-	-	-	-	-	-	49%

^a^ Prevalence of anxiety disorders assessed using a diagnostic interview method. ^b^ Prevalence of anxiety disorders assessed using a self-report screening scale. (-) Anxiety disorder not assessed.

**Table 5:** Prevalence of comorbid mood disorders in misophonia.

	Mood Disorders								
	Depression Symptomology	Major Depressive Disorder	Major Depressive Episode	Persistent Depressive Disorder	Dysthymic Disorder	Premenstrual Dysphoric Disorder	Suicide Behavior Disorder	Bipolar Disorder	Any Mood Disorder
**Abramovitch et al. (2024) ^a^** **(*N* = 32)**	-	3.1%	-	-	-	-	-	-	-
**Begenen et al. (2023) ^a^** **(*N* = 39)**	-	17.9%	-	-	5.1%	15.4%	-	-	-
**Cassiello-Robbins et al. (2021) ^a^** **(*N* = 49)**	-	18.4%	-	-	-	-	-	-	-
**Erfanian et al. (2019) ^a^** **(*N* = 52)**	-	9.61%	-	-	5.76%	-	1.92%	-	-
**Frank and McKay. (2019) ^a^** **(*N* = 18)**	-	11%	-	-	-	6%	-	-	-
**Guzick et al. (2023) ^a^** **(*N* = 102)**	-	-	15%	-	-	-	-	-	-
**Jager et al. (2020) ^a^** **(*N* = 575)**	-	6.8%	-	-	1.7%	-	-	1.2%	-
**Lewin et.al (2023) ^a^** **(*N* = 46)**	-	-	8.5%	-	4.3%	-	-	-	-
**Neacsiu et al. (2024) ^a^** **(*N* = 29)**	-	-	-	-	-	-	-	-	13.8%
**Rosenthal et al. (2022) ^a^** **(*N* = 207)**	-	6.8%	-	7.7%	-	1.9%	-	1.0%	-
**Schröder et al. (2013) ^a^** **(*N* = 42)**	-	7.1%	-	-	-	-	-	-	-
**Schröder et al. (2017) ^a^** **(*N* = 90)**	-	1.1%	-	-	2.2%	-	-	1.1%	-
**Siepsiak et al. (2022) ^a^** **(*N* = 71)**	-	11.9%	37.3%	-	-	-	20%	10.5% (w/psychotic features 4.5%)	-
**Yektatalab et al. (2022) ^b^** **(*N* = 93)**	9.7%	-	-	-	-	-	-	-	-

^a^ Prevalence of mood disorders assessed using a diagnostic interview method. ^b^ Prevalence of mood disorders assessed using a self-report screening scale. (-) Mood disorder not assessed.

**Table 6:** Prevalence of comorbid anxiety and trauma-related disorders in misophonia.

	Anxiety and Trauma Related Disorders									
	Any Anxiety Disorder	GAD	Social Anxiety	Panic	Agoraphobia	Separation Anxiety Disorder	Specified Phobia	PTSD	Adjustment Disorder	Any Trauma/Stress Disorder
**Abramovitch et al. (2024) ^a^** **(*N* = 32)**	18.8%	3.1%	3.1%	6.3%	3.2%	-	-	3.1%	-	-
**Begenen et al. (2023) ^a^** **(*N* = 39)**	59%	12.8%	17.9%	10.3%	25.6%	-	12.8%	-	-	-
**Cassiello-Robbins et al. (2021) ^a^** **(*N* = 49)**	-	32.7%	10.2%	-	-	-	-	18.4%	-	-
**Erfanian et al. (2019) ^a^** **(*N* = 52)**	-	1.9%	5.7%	1.9%	1.9%	-	-	15.4%	-	-
**Frank and McKay. (2019) ^a^** **(*N* = 18)**	-	17%	11%	6%	11%	-	-	-	-	-
**Guetta et al.** **(2024) ^a^**	-	-	-	-	-	-	-	3.5%	2.8%	5.5%
**Guzick et al. (2023) ^a^** **(*N* = 102)**	-	27%	30%	-	-	-	-	-	-	-
**Jager et al. (2020)^a^** **(*N* = 575)**	0.2%	1.0%	1.2%	0.9%	-	0.2%	-	1.7%	-	-
**Lewin et.al (2023) ^a^** **(*N* = 46)**	59.6%	36.2%	29.8%	-	-	2.1%	12.9%	6.4%	-	-
**Neacsiu et al. (2024) ^a^** **(*N* = 29)**	69%	-	-	-	-	-	-	-	-	10.3%
**Rosenthal et al. (2022) ^a^** **(*N* = 207)**	-	24.6%	30.9%	2.9%	10.1%	-	13.5%	2.9%	-	-
**Schröder et al. (2013) ^a^** **(*N* = 42)**	-	-	-	2.4%	-	-	-	-	-	-
**Siepsiak et al. (2022) ^a^** **(*N* = 71)**	-	14.9%	13.4%	19.4%	4.5%	-	-	11.9%	-	-
**Yektatalab et al. (2022) ^b^** **(*N* = 93)**	8.6%	-	-	-	-	-	-	-	-	-

^a^ Prevalence of anxiety and trauma-related disorders assessed using a diagnostic interview method. ^b^ Prevalence of anxiety and trauma-related disorders assessed using a self-report screening scale. (-) Anxiety and trauma-related disorder not assessed.

**Table 7:** Prevalence of comorbid obsessive-compulsive related disorders in misophonia.

	Obsessive-Compulsive Related Disorders					
	OCD	Body Dysmorphia	Excoriation	Hoarding	Trichotillomania	Onychophagia
**Abramovitch et al. (2024) ^a^** **(*N* = 32)**	9.4%	-	-	-	-	-
**Begenen et al. (2023) ^a^** **(*N* = 39)**	-	2.6%	5.1%	12.8%	-	17.9%
**Cassiello-Robbins et al. (2021) ^a^** **(*N* = 49)**	6.1%	-	-	-	-	-
**Erfanian et al. (2019) ^a^** **(*N* = 52)**	11.5%	-	-	-	-	-
**Frank and McKay. (2019) ^a^** **(*N* = 18)**	-	-	6%	-	-	-
**Guzick et al. (2023) ^a^** **(*N* = 102)**	8%	-	-	-	-	-
**Jager et al. (2020)^a^** **(*N* = 575)**	2.8%	-	-	-	1.9%	-
**Lewin et.al (2023) ^a^** **(*N* = 46)**	2.1%	-	-	-	-	-
**Rosenthal et al. (2022) ^a^** **(*N* = 207)**	8.2%	-	5.3%	1.9%	1.9%	-
**Schröder et al. (2013) ^a^** **(*N* = 42)**	2.4%	-	2.4%	-	4.8%	-
**Schröder et al. (2017) ^a^** **(*N* = 90)**	3.3%	1.1%	5.5%	-	-	-
**Siepsiak et al. (2022) ^a^** **(*N* = 71)**	6%	-	-	-	-	-
**Yektatalab et al. (2022) ^b^** **(*N* = 93)**	39.8%	-	-	-	-	-

^a^ Prevalence of obsessive-compulsive related disorder assessed using a diagnostic interview method. ^b^ Prevalence of obsessive-compulsive related disorder assessed using a self-report screening scale. (-) obsessive-compulsive disorder not assessed.

**Table 8:** Prevalence of comorbid eating disorders in misophonia.

	Eating Disorders				
	Any Eating Disorder	Anorexia	Bulimia	Binge Eating	Other
**Abramovitch et al. (2024) ^a^** **(*N* = 32)**	6.3%	3.1%	3.1%	3.1%	-
**Begenen et al. (2023) ^a^** **(*N* = 39)**	-	-	-	15.4%	-
**Erfanian et al. (2019) ^a^** **(*N* = 52)**	-	9.6%	7.7%	-	-
**Jager et al. (2020) ^a^** **(*N* = 575)**	0.7%	-	-	-	-
**Neacsiu et al. (2024) ^a^** **(*N* = 29)**	3.4%	-	-	-	-
**Rosenthal et al. (2022) ^a^** **(*N* = 207)**	-	-	1.4%	0.5%	0.5%
**Siepsiak et al. (2022) ^a^** **(*N* = 71)**	-	14.9%	11.9%	-	-

^a^ Prevalence of eating disorders assessed using a diagnostic interview method. (-) Eating disorder not assessed.

**Table 9:** Prevalence of comorbid substance use disorders in misophonia.

	Substance Use Disorders				
	Substance Dependence	Alcohol Dependence	Cannabis Dependence	Cocaine Dependence	Other
**Abramovitch et al. (2024)** **(*N* = 32)**	9.4%	-	-	-	-
**Erfanian et al. (2019) ^a^** **(*N* = 52)**	1.92%	3.84%	-	-	-
**Frank and McKay. (2019) ^a^** **(*N* = 18)**	-	11%	-	-	-
**Jager et al. (2020) ^a^** **(*N* = 575)**	-	1.0%	0.5%	-	-
**Rosenthal et al. (2022) ^a^** **(*N* = 207)**	-	1.9%	1.4%	0.5%	0.5%

^a^ Prevalence of substance dependence disorder assessed using a diagnostic interview method. (-) Substance dependence disorder not assessed.

**Table 10:** Prevalence of comorbid neurodevelopmental disorders in misophonia.

Neurodevelopmental Disorders								
	Attention Deficit Disorder			Autism Spectrum Disorder		Tic Disorder		
	ADHD	ADD	Combined	Autistic Disorder	Pervasive Development Disorder	Tic disorder	Gilles de la Tourette	Chronic Motor Disorder
**Abramovitch et al. (2024) ^a^** **(*N* = 32)**	15.6%	-	-	-	-	-	-	-
**Begenen et al. (2023) ^a^** **(*N* = 39)**	20.5%	-	-	-	-	5.1%	-	-
**Frank and McKay. (2019) ^a^** **(*N* = 18)**	6%	-	-	-	-	-	-	-
**Guzick et al. (2023) ^a^** **(*N* = 102)**	21%	-	-	-	-	13%	-	--
**Jager et al. (2020) ^a^** **(*N* = 575)**	1.7%	3.3%	0.3%	1.2%	1.2%		0.3%	0.5%
**Lewin et.al (2023) ^a^** **(*N* = 46)**	14.9%	-	-	4.3%	-	6.4%	-	-
**Rinaldi et al. (2022) ^b^** **(*N* = 126)**	-	-	-	21.4% *	-	-	-	-
**Rinaldi et al. (2022) ^b^** **(*N* = 15)**	-	-	-	12.5% **	-	-	-	-
**Rosenthal et al. (2022) ^a^** **(*N* = 207)**	17.9%	-	-	2.4%	-	-	-	--
**Schröder et al. (2013) ^a^** **(*N* = 42)**	4.8%	-	-	-	-	-	4.8%	-
**Schröder et al. (2017) ^a^** **(*N* = 90)**	4.4%	-	-	-	-	-	-	-
**Siepsiak et al. (2023) ^a^** **(*N* = 45)**	2.4%	16.7%	7.1%	-	-	18.2%	-	-

^a^ Disorder assessed using a diagnostic interview method. ^b^ Disorder assessed using a self-report screening scale. * Population: adults with misophonia. ** Population: Children with misophonia. (-) Neurodevelopmental disorder not assessed.

**Table 11:** Prevalence of comorbid personality disorders in misophonia.

	Personality Disorder						
	Obsessive-Compulsive	Borderline	Avoidant	Dependent	Antisocial	Narcissistic	Paranoid
**Cassiello-Robbins et al. (2020) ^a^** **(*N* = 49)**	10.2%	12.2%	8.2%	-	-	-	-
**Jager et al. (2020) ^a^** **(*N* = 575)**	2.4%	1.7%	0.5%	0.2%	0.2%	-	-
**Rosenthal et al. (2022) ^a^** **(*N* = 207)**	5.8%	2.9%	2.9%	0.5%	-	0.5%	0.5%
**Schröder et al. (2013) ^a^** **(*N* = 42)**	52.4%	-	-	-	-	-	-

^a^ Prevalence of personality disorder assessed using a diagnostic interview method. (-) Personality disorder not assessed.

## Data Availability

No new data were created or analyzed in this study. Data sharing is not applicable to this article.

## Appendix A

Search Strategy

PubMed: ((((((((((((Hyperacusis) OR (Misophonia)) OR (“Auditory hypersensitivity”)) OR (“ Auditory Sensitivity”)) OR (“Noise Sensitivity”)) OR (“sound sensitivity”)) OR (“Sound Intolerance”)) OR (“decreased sound tolerance”)) OR (“aversive sound*”)) OR (“trigger sound*”)) OR (“Selective Sound Sensitivity Syndrome”)) AND (((((((Psychiatric symptom) OR (Psychiatric comorbidity)) OR (Psychiatric disorder)) OR (Mental disorder)) OR (Psychopathology)) OR (Psychiatric Illness)) OR (Mental Illness)))

PsycInfo: (Any Field: (Hyperacusis OR Any Field: Misophonia OR Any Field: “Auditory hypersensitivity” OR Any Field: “ Auditory Sensitivity” OR Any Field: “Noise Sensitivity” OR Any Field: “sound sensitivity” OR Any Field: “Sound Intolerance” OR Any Field: “decreased sound tolerance” OR Any Field: “aversive sound*” OR Any Field: “trigger sound*” OR Any Field: “Selective Sound Sensitivity Syndrome”) AND (Any Field: Psychiatric symptom OR Any Field: Psychiatric comorbidity OR Any Field: Psychiatric disorder OR Any Field: Mental disorder OR Any Field: Psychopathology OR Any Field: Psychiatric Illness OR Any Field: Mental Illness)

Scopus: (TITLE-ABS-KEY (hyperacusis OR misophonia OR “Auditory Hypersensitivity” OR “Auditory Sensitivity” OR “Sound sensitivity” OR “Sound Intolerance” OR “Decreased sound tolerance” OR “Aversive sound*” OR “Trigger sound*” OR “Selective Sound Sensitivity Syndrome”) AND TITLE-ABS-KEY (“Psychiatric symptom*” OR “Psychiatric comorbidit*” OR “Psychiatric disorder*” OR “Mental disorder*” OR “Psychopathology” OR “psychiatric illness” OR “mental illness”))

Web Of Science: (Hyperacusis OR Misophonia OR “Auditory Hypersensitivity” OR “ Auditory Sensitivity” OR “Noise Sensitivity” OR “sound sensitivity” OR “Sound Intolerance” OR “decreased sound tolerance” OR “aversive sound*” OR “trigger sound*” OR “Selective Sound Sensitivity Syndrome”) (Topic) and (“Psychiatric symptom*” OR “Psychiatric comorbidit*” OR “Psychiatric disorder*” OR “Mental disorder*” OR Psychopathology OR “Psychiatric Illness” OR “Mental Illness”) (Topic)

## Appendix B

**JBI for Prevalence Data**										
**Author and Year**	**Appropriate Sample Frame**	**Appropriate Sampling of Participants**	**Adequate Sample Size**	**Detailed Description of Subjects and the Setting**	**Data Analysis: Sufficient Coverage of the Identified Sample**	**Valid Methods for Identification of the Condition**	**Standard and Reliable Measure of the Condition for All Participants**	**Appropriate Statistical Analysis**	**Adequate Response Rate or the Appropriate Management of Low Response Rate**	**Total Score**
Aazh and Moore (2017)	N	N	N	Y	N	Y	Y	Y	Y	5/9
Begenen et al. (2023)	N	N	N	Y	Y	Y	Y	Y	Y	6/9
Cassiello-Robbins et al. (2020)	N	N	N	Y	N	Y	Y	Y	Y	5/9
Erfanian et al. (2019)	N	N	N	Y	Y	Y	Y	Y	Y	6/9
Goebel and Floetzinger (2008)	N	Y	N	Y	Y	Y	Y	Y	Y	7/9
Guetta et al. (2024)	N	N	Y	Y	Y	Y	Y	Y	Y	7/9
Guzick et al. (2023)	N	N	Y	Y	Y	Y	Y	Y	Y	7/9
Lewin et.al (2023)	N	N	N	Y	Y	Y	Y	Y	Y	6/9
Jager et al. (2020)	N	Y	Y	Y	N	Y	N	Y	Y	6/9
Jüris et al. (2012)	N	N	N	Y	Y	Y	Y	Y	Y	6/9
Rosenthal at al. (2022)	N	N	Y	Y	N	Y	Y	Y	Y	6/9
Schröder et al. (2013)	N	N	N	Y	Y	Y	Y	Y	N	5/9
Siepsiak et al. (2022)	N	Y	N	Y	Y	Y	Y	Y	Y	7/9
Smit et al. (2021)	N	Y	Y	Y	Y	Y	Y	Y	Y	8/9
Theodoroff et al. (2024)	N	Y	Y	N	Y	Y	Y	Y	Y	7/9
Yektatalab et al. (2022)	N	N	N	Y	Y	Y	Y	Y	Y	6/9

**JBI for Case Control Studies**											
**Author and Year**	**Comparable Groups**	**Case and Controls Matched Properly**	**Same Criteria Used for Identification**	**Exposure Measured in a Standard, Valid and Reliable Way**	**Exposure Measured in the Same Way for Cases and Controls**	**Confounding Factors Identified**	**Strategies to Deal with Confounding Factors Stated**	**Outcomes Assessed in a Standard, Valid and Reliable Way for Cases and Controls**	**Appropriate Exposure Period**	**Appropriate Statistical Analysis**	**Total**
Abramovitch et al. (2024)	N	N	Y	Y	Y	N	N	Y	Y	Y	6/10
Rinaldi et al. (2022) ^1^	N	N	N	Y	Y	N	N	Y	Y	Y	5/10
Rinaldi et al. (2022) ^2^	Y	Y	Y	Y	Y	N	N	Y	Y	Y	8/10
Siepsiak et al. (2023)	Y	Y	Y	Y	Y	N	N	Y	Y	Y	8/10
^1^ Population: adults with misophonia. ^2^ Population: Children with misophonia.											

**JBI for RCT**														
**Author and Year**	**Was True Randomization Used for Assignment of Participants to Treatment Groups?**	**Was Allocation Concealed?**	**Were Treatment Groups Similar at the Baseline?**	**Were Participants Blind to Treatment?**	**Were Those Delivering Treatment Blind to Treatment Assignment?**	**Were Outcomes Assessors Blind to Treatment Assignment?**	**Were Treatment Groups Treated Identically Other Than the Intervention of Interest?**	**Was Follow up Complete?**	**Were Participants Analyzed in the Groups to Which They Were Randomized?**	**Were Outcomes Measured in the Same Way for Treatment Groups?**	**Were Outcomes Measured in a Reliable Way?**	**Was Appropriate Statistical Analysis Used?**	**Was the Trial Design Appropriate, and Any Deviations from the Standard RCT Design Accounted for in?**	**Total**
Frank and McKay (2019)	Y	N	Y	N	N	N	Y	Y	Y	Y	Y	Y	N	8/13
Neacsiu et al.(2024)	N	N	Y	Y	Y	N	Y	Y	Y	Y	Y	Y	Y	10/13
Schröder et al. (2017)	N	N	Y	N	N	N	Y	Y	Y	Y	Y	Y	N	7/13
